# Precision of Corneal Thickness Measurements Obtained Using the Scheimpflug-Placido Imaging and Agreement with Ultrasound Pachymetry

**DOI:** 10.1155/2015/328798

**Published:** 2015-02-24

**Authors:** Jinhai Huang, Giacomo Savini, Chengfang Wang, Weicong Lu, Rongrong Gao, Yuanguang Li, Qinmei Wang, Yune Zhao

**Affiliations:** ^1^School of Ophthalmology and Optometry, Eye Hospital, Wenzhou Medical University, 270 West Xueyuan Road, Wenzhou, Zhejiang 325027, China; ^2^Key Laboratory of Vision Science, Ministry of Health, Wenzhou, Zhejiang, China; ^3^G.B. Bietti Eye Foundation, IRCCS, Rome, Italy

## Abstract

*Purpose.* To assess the reliability and comparability of measuring central corneal thickness (CCT) and thinnest corneal thickness (TCT) using a new Scheimpflug-Placido analyzer (TMS-5, Japan) and ultrasound (US) pachymetry.* Methods.* Seventy-six healthy subjects were prospectively measured 3 times by 1 operator using the TMS-5, 3 additional consecutive scans were performed by a second operator, and ultrasound (US) pachymetry measurements were taken. The test-retest repeatability (TRT), coefficient of variation (CoV), and intraclass correlation coefficient (ICC) were calculated to evaluate intraoperator repeatability and interoperator reproducibility. Agreement among the devices was assessed using Bland-Altman plots and 95% limits of agreement (LoA).* Results.* The intraoperators TRT and CoV were <19 *μ*m and 2.0%, respectively. The interoperators TRT and CoV were <12 *μ*m and 1.0%, respectively, and ICC was >0.90. The mean CCT and TCT measurements using the TMS-5 were 15.97 *μ*m (95% LoA from −26.42 to −5.52 *μ*m) and 20.32 *μ*m (95% LoA from −30.67 to −9.97 *μ*m) smaller, respectively, than those using US pachymetry.* Conclusions.* The TMS-5 shows good repeatability and reproducibility for measuring CCT and TCT in normal subjects but only moderate agreement with US pachymetry results. Caution is warranted before using these techniques interchangeably.

## 1. Introduction

Precise measurement of central corneal thickness (CCT) is important when planning excimer laser surgery and diagnosing glaucoma and other corneal diseases [1, 2]. An overestimation of CCT and the thinnest corneal thickness (TCT) before corneal refractive surgery could lead to corneal stroma overablation, especially in eyes with a high degree of myopia or borderline corneal thickness or that require enhancement surgeries, and can also increase the risk of keratectasia [3]. Accurate CCT measurements are also useful when monitoring corneal disorders, such as keratoconus and contact lens-related complications [4]; therefore, instruments that can evaluate CCT and TCT are now mandatory in clinical practice.

Several technologies for measuring corneal thickness are available, the most common of which, for many years, has been ultrasound (US) pachymetry, which is regarded as the gold standard because of its low cost, compact design, ease of use, and high repeatability; however, US pachymetry requires direct corneal contact, which causes an indentation of the cornea and could lead to false results [5]. Its reliability can also be limited by the operator's skill and experience in manually placing the US pachymetry probe onto the center of the cornea [6, 7]. Contact with a patient's eyes could cause discomfort or even damage the corneal epithelium [8]. These limitations led to the development of different, more sophisticated, noncontact technologies, such as Scheimpflug imaging. Rotating Scheimpflug cameras include the Pentacam (Oculus, Wetzlar, Germany), Sirius (Costruzione Strumenti Oftalmici, Florence, Italy), Galilei (Ziemer Ophthalmology, Port, Switzerland), and the Scheimpflug-Placido topographic modeling system (TMS-5, Tomey Corporation, Nagoya, Japan). Several studies have shown that the Pentacam, Sirius, and Galilei provide reliable CCT and TCT measurements [9, 10] and that these devices offer good repeatability and reproducibility comparable to US pachymetry in both normal and postsurgery eyes. The TMS-5, the newest among the above-mentioned Scheimpflug cameras, has received little attention because it has been on the market for the least amount of time. Only 2 studies have investigated this device. Guilbert et al. [11] found that the TMS-5 provides excellent repeatability and lower mean CCT values compared to those of the Orbscan II (Bausch & Lomb, Rochester, NY, USA) and US pachymetry. Similarly, Savini et al. [12] found that the mean CCT measurements using the TMS-5 are lower compared to those of Sirius and Pentacam.

The aim of this study was to comprehensively assess the intraoperator repeatability and interoperator reproducibility of CCT and TCT measurements taken with the TMS-5 and to compare these measurements with those provided by US pachymetry in normal eyes.

## 2. Subjects and Methods

### 2.1. Subjects

Seventy-six healthy subjects (47 female) with a mean age of 26.47 ± 4.16 years (range, 21 to 49 years) were prospectively recruited at the Eye Hospital of Wenzhou Medical University, China. The mean manifest spherical equivalent refraction was −3.78 ± 2.48 diopters (D) (range, 1.00 to −10.00 D). One eye of each subject was randomly selected. The exclusion criteria were ocular pathology, corrected vision <20/20, intraocular pressure >21 mmHg, corneal astigmatism >2.0 D, history of ophthalmic surgery, and recent contact lens wear (rigid contact lens within 4 weeks and soft contact lens within 2 weeks).

Before the experiment, all eyes underwent a comprehensive ophthalmic examination, comprising refraction, slit-lamp microscopy, noncontact tonometry, corneal topography (Keratron, Optikon 2000 SpA, Rome, Italy), and ophthalmoscopy. Each subject was informed of the study's purpose and gave his or her informed written consent to participate before being enrolled in the study. The study methods adhered to the tenets of the Declaration of Helsinki for the use of human participants in biomedical research and were approved by the research review board at the Eye Hospital of Wenzhou Medical University.

### 2.2. Instrument

The TMS-5 uses a combination of a rotating Scheimpflug camera and a wide-angle Placido ring topographer (with 31 rings). It first captures the ring topography and then the 32 slit-scan images. The 2 acquisition steps are separate, and the data are merged at the end of the examination.

### 2.3. Measurement Procedures

All eyes were measured without pupil dilation between 10:00 a.m. and 5:00 p.m. to minimize the effects of diurnal variation in corneal thickness and shape [13]. In the first part of the study, the use of the precision of the TMS-5 was investigated. Each subject was assessed in a dim room by 2 well-trained operators. All subjects were positioned correctly and asked to fixate on a target without blinking during each scan. Each subject was then instructed to blink once before the scan so as to spread a smooth tear film over the cornea. After completing each measurement, each subject was asked to relax and the system was realigned for the next measurement. Three consecutive measurements were taken by each operator, which were used to study intraoperator repeatability. The values of the 3 successive measurements using the TMS-5 were then averaged to obtain the mean and the differences between the mean values obtained by the 2 operators were used to assess interoperator reproducibility.

In the second part of the study, agreement on corneal thickness as measured using both the TMS-5 and US pachymetry was investigated. After the noncontact examination using the TMS-5, the cornea was anesthetized with 0.5% proparacaine hydrochloride (Alcaine). The SP-3000 ultrasound pachymeter (Tomey Corporation, Nagoya, Japan) was precalibrated before the study. The US velocity was set at 1640 m/s, and the probe was aligned as perpendicularly as possible with the center of the cornea. Five readings were obtained, and the highest and the lowest values were excluded; the remaining 3 measurements were averaged. This value was then compared with those of the CCT and TCT measured by the TMS-5.

### 2.4. Statistical Analyses

All data were analyzed using SPSS version 21.0 for Windows (IBM Corporation, Armonk, NY, USA). The results were presented as the mean ± standard deviation (SD). Kolmogorov-Smirnov tests were used to check dataset distributions, and the results indicated that the data were normally distributed (*P* > 0.05). A *P* value of ≤0.05 was considered statistically significant.

To determine the intraoperator repeatability of TMS-5, within-subject standard deviation (Sw), test-retest repeatability (TRT), within-subject coefficient of variation (CoV), and intraclass correlation coefficients (ICCs) were calculated for the 3 repeated measurements obtained by the first and second operators. TRT was defined as 2.77 Sw, which means an interval within which 95% of the difference between measurements is expected to lie [14]. CoV was calculated as the ratio of Sw to the overall mean. A lower CoV is associated with higher repeatability. The closer the ICC is to 1.0, the better the measurement consistency is. To assess interoperator reproducibility, the mean of the 3 readings from each operator was first calculated for each device, and then interoperator Sw, 2.77 Sw, CoV, and ICC were calculated.

Bland-Altman plots were used to analyze the agreement between pairs of measurements [15]. The difference between the measurements taken using the 2 devices was plotted against their means, and the 95% LoA and 95% confidence intervals (CIs) were determined.

## 3. Results

### 3.1. Intraoperator Repeatability

CCT and TCT measurements taken with the TMS-5 showed good intraoperator repeatability (Table 1). The intraoperator TRT and CoV were less than 19 *μ*m and 2.0%, respectively, and the ICC was >0.90.

### 3.2. Interoperator Reproducibility

Interoperator reproducibility was reported as well using the CCT and TCT measurements taken with the TMS-5. The interoperator TRT and CoV were <12 *μ*m and 1.0%, respectively, and the ICC was >0.90 (Table 2).

### 3.3. Comparison between Devices

**Figure 1:**
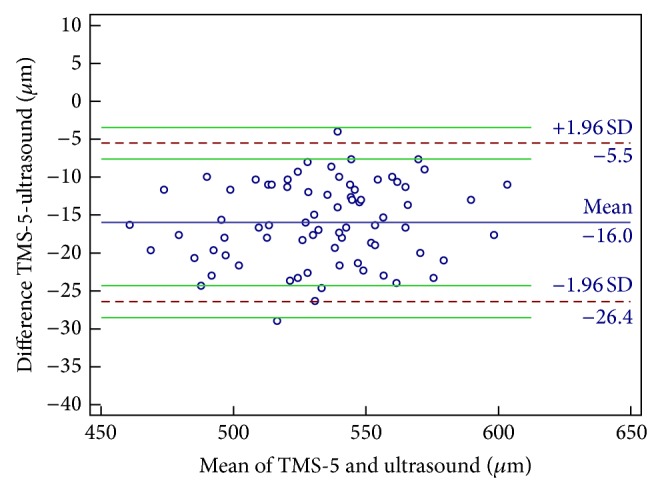
Bland-Altman plots of agreement in the central corneal thickness measurement between the Scheimpflug-Placido topographic modeling system (TMS-5) and ultrasound pachymetry. The blue solid line indicates the mean difference (bias). The dashed lines represent the 95% limits of agreement. The upper and lower green solid lines represent the 95% limits of agreement.

**Figure 2:**
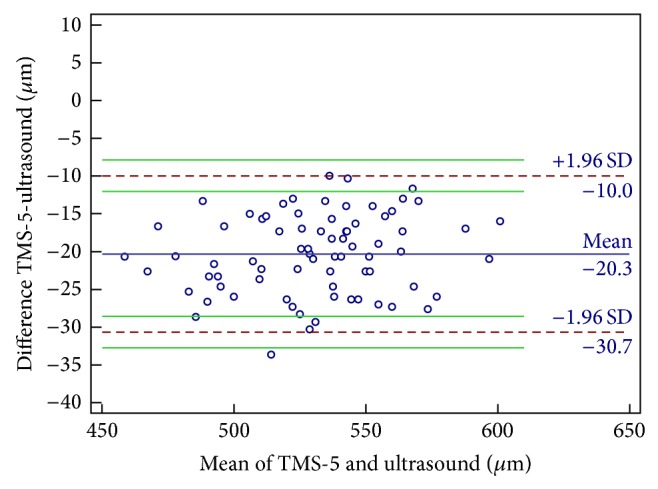
Bland-Altman plots of agreement in the thinnest corneal thickness measurement between the Scheimpflug-Placido topographic modeling system (TMS-5) and ultrasound pachymetry. The blue solid line indicates the mean difference (bias). The dashed lines represent the 95% limits of agreement. The upper and lower green solid lines represent the 95% limits of agreement.

The mean CCT and TCT as measured using the TMS-5 were 525.22 ± 30.41 and 520.87 ± 30.49 *μ*m, respectively. The mean CCT measurement using US pachymetry was 541.19 ± 29.62 *μ*m. The mean CCT using the TMS-5 was significantly lower than that using US pachymetry (15.97 *μ*m). There were also significant differences between the TCT measurements using the TMS-5 and US pachymetry, with the mean TMS-5 TCT measurements being −20.32 *μ*m lower than those of the US pachymetry (Table 3). In terms of agreement between the 2 devices, the CCT and TCT measurements showed broad 95% LoA, which means moderate-to-poor agreement (Figures 1 and 2). A fixed bias was detected between both CCT and TCT using these 2 devices.

## 4. Discussion

Accurate quantitative measurements of CCT and TCT provide valuable information during clinical examinations. US pachymetry is still the most widely used technique to measure CCT and TCT; however, it has moderate resolution and precision, depends on operator skills, and causes discomforting to the patient [16, 17]; consequently, noncontact devices represent a more desirable alternative and have been widely applied in both clinical practice and research settings, although there remains an issue with their precision. It is important to compare the measurements obtained by these recently introduced imaging devices with those of the old standard devices. The agreement of the CCT and TCT measurements obtained by the TMS-5 with US pachymetry and the reproducibility of the TMS-5 measurements were evaluated. The data suggest that the TMS-5 shows good repeatability and reproducibility for CCT and TCT in normal subjects but moderate agreement with US pachymetry because it underestimates the measurements taken by US pachymetry.

Several studies have investigated the other rotating Scheimpflug cameras (i.e., Pentacam, Sirius, and Galilei) and found good repeatability and reproducibility of CCT and TCT in both healthy and post-refractive-surgery eyes. Nam et al. [7] reported a TRT and CoV of CCT of 10.0 mm and 0.67%, respectively, using the Pentacam. Hernández-Camarena et al. [18] demonstrated strong repeatability of CCT measurements using the Sirius, and similar results were reported by Maresca et al. [19]. Some studies also showed good repeatability and reproducibility from the Galilei. Huang et al. [20] found low intraoperator and interoperator variation in the Galilei G2 measurements with an ICC of 0.99 in healthy eyes, which means it has good concordance and high reproducibility. Savini et al. [10] reported high repeatability of CCT and TCT measurements using Galilei; the CoV of each was 0.40% and 0.33%, respectively, while the TRT of each was 5.97 and 4.78 mm, respectively. Wang et al. [9] reported a low CCT CoV of 0.25%. Recently, Guilbert et al. [11] found that the repeatability of the TMS-5 was excellent with an ICC of 0.953, which is comparable to that of our results.

On comparing CCT and TCT using Scheimpflug imaging with those using US pachymetry, most studies revealed close agreement among the methods. In a study of healthy eyes, Sedaghat et al. [21] reported close agreement between the Pentacam and US pachymetry. O'Donnell and Maldonado-Codina [22] found that the Pentacam provided measurements that were slightly but systematically lower than those obtained by US pachymetry but still showed close agreement between the 2 methods. Similar results were reported by Al-Mohtaseb et al. [23], who found that Pentacam measurements agree with those of US pachymetry; CCT measurements by the Pentacam were larger than those of US pachymetry by 8.2 *μ*m. On the contrary, a recent study by Guilbert et al. [11] reported significantly smaller CCT measurements with the TMS-5 than with other systems, such as the Orbscan II and US pachymetry, which is in accordance with our results. They suggest that the discrepancies were most likely to be clinically significant and that the readings obtained with the TMS-5 and the other 2 devices could not be used interchangeably.

Our results reveal only moderate agreement between the TMS-5 system and US pachymetry; therefore, caution is warranted before using these instruments interchangeably for measuring normal eyes. The mean CCT and TCT using the TMS-5 underestimate those of US pachymetry by an average of 15.97 and 20.32 *μ*m, respectively. These differences are clinically significant and are not consistent with most of those in previous studies that compared other Scheimpflug devices with US pachymetry in healthy patients. Chen et al. [24] and Al-Mezaine et al. [25] found that the Pentacam CCT measurements were larger than those of US pachymetry by 5.7 and 8.2 *μ*m, respectively. On the other hand, according to O'Donnell and Maldonado-Codina [22] and Lackner et al. [26], the Pentacam gave CCT results that were lower by 6.0 and 10.0 *μ*m, respectively, compared with those of the US pachymetry. Jahadi Hosseini et al. [27] found that the CCT measurements obtained with the Galilei were larger than those obtained with US pachymetry by 11.04 *μ*m. Similar results were reported by Jorge et al. [28] for the CCT measurement obtained with the Sirius, which was 4.68 *μ*m larger than that provided by US pachymetry. Savini et al. [12] found that the mean CCT measurements taken by Sirius were 24.0 *μ*m larger than those taken with the TMS-5 in healthy eyes. Several reasons could explain the differences in measurements between the TMS-5 and US pachymetry. First, because measurements by the Scheimpflug camera and the Placido disk topographer are separated, there might be some problems during the merging procedure if the measurements are not taken along the same axis. Second, the accuracy of US pachymetry might be biased by the experience of the operator. Third, the examination of US pachymetry requires the use of anesthetic, which could cause corneal swelling and lead to measurements that erroneously report a greater thickness [29].

This study was limited in which only healthy patients were investigated; therefore, our results cannot be applied to other conditions, such as keratoconus and post-refractive surgery.

In conclusion, the TMS-5 showed good intraoperator repeatability and interoperator reproducibility of CCT and TCT measurements in healthy eyes but showed only moderate agreement with US pachymetry. The CCT and TCT measurements obtained by the TMS-5 and US pachymetry cannot be considered interchangeable in eyes with normal corneal thickness.

## Figures and Tables

**Table 1:** Intraoperator repeatability of central corneal thickness (CCT) and the thinnest corneal thickness (TCT) readings using the Scheimpflug-Placido topographic modeling system (TMS-5).

Parameters	Mean ± SD (*μ*m)	Sw (*μ*m)	2.77 Sw (*μ*m)	CoV (%)	ICC (95% CI)
1st observer					
CCT	525.22 ± 30.41	6.51	18.05	1.24	0.955 (0.936 to 0.970)
TCT	520.87 ± 30.49	6.60	18.27	1.27	0.955 (0.934 to 0.970)
2nd observer					
CCT	525.67 ± 29.62	6.32	17.50	1.20	0.956 (0.935 to 0.971)
TCT	521.43 ± 29.64	6.39	17.70	1.23	0.955 (0.934 to 0.970)

SD: standard deviation; Sw: within-subject standard deviation; CoV: coefficient of variation; ICC: intraclass correlation coefficient; CI: confidence interval.

**Table 2:** Interoperator reproducibility of central corneal thickness (CCT) and the thinnest corneal thickness (TCT) readings by the Scheimpflug-Placido topographic modeling system (TMS-5).

Parameters	Sw (*μ*m)	2.77 Sw (*μ*m)	CoV (%)	ICC (95% CI)
CCT	4.09	11.32	0.78	0.981 (0.971 to 0.988)
TCT	4.01	11.11	0.77	0.982 (0.972 to 0.989)

Sw: within-subject standard deviation; CoV: coefficient of variation; ICC: intraclass correlation coefficient; CI: confidence interval.

**Table 3:** Comparison of central corneal thickness (CCT) and the thinnest corneal thickness (TCT) readings by the Scheimpflug-Placido topographic modeling system (TMS-5) and ultrasound pachymetry.

Parameters	Mean difference ± SD	*P* value	Lower 95% LoA (95% CI)	Upper 95% LoA (95% CI)
CCT (*μ*m)	−15.97 ± 5.33	<0.001	−26.42 (−28.53 to −24.33)	−5.52 (−7.63 to −3.42)
TCT (*μ*m)	−20.32 ± 5.28	<0.001	−30.67 (−32.75 to −28.58)	−9.97 (−12.06 to −7.89)

SD: standard deviation; LoA: limits of agreement; CI: confidence interval.

## References

[1] Gordon M. O., Beiser J. A., Brandt J. D. (2002). The Ocular Hypertension Treatment Study: baseline factors that predict the onset of primary open-angle glaucoma. *Archives of Ophthalmology*.

[2] Price F. W., Koller D. L., Price M. O. (1999). Central corneal pachymetry in patients undergoing laser in situ keratomileusis. *Ophthalmology*.

[3] Randleman J. B., Woodward M., Lynn M. J., Stulting R. D. (2008). Risk assessment for ectasia after corneal refractive surgery. *Ophthalmology*.

[4] Gromacki S. J., Barr J. T. (1994). Central and peripheral corneal thickness in keratoconus and normal patient groups. *Optometry and Vision Science*.

[5] Izatt J. A., Hee M. R., Swanson E. A. (1994). Micrometer-scale resolution imaging of the anterior eye in vivo with optical coherence tomography. *Archives of Ophthalmology*.

[6] Miglior S., Albe E., Guareschi M., Mandelli G., Gomarasca S., Orzalesi N. (2004). Intraobserver and interobserver reproducibility in the evaluation of ultrasonic pachymetry measurements of central corneal thickness. *British Journal of Ophthalmology*.

[7] Nam S. M., Im C. Y., Lee H. K., Kim E. K., Kim T.-I., Seo K. Y. (2010). Accuracy of RTVue optical coherence tomography, pentacam, and ultrasonic pachymetry for the measurement of central corneal thickness. *Ophthalmology*.

[8] Kawana K., Tokunaga T., Miyata K., Okamoto F., Kiuchi T., Oshika T. (2004). Comparison of corneal thickness measurements using Orbscan II, non-contact specular microscopy, and ultrasonic pachymetry in eyes after laser in situ keratomileusis. *British Journal of Ophthalmology*.

[9] Wang L., Shirayama M., Koch D. D. (2010). Repeatability of corneal power and wavefront aberration measurements with a dual-Scheimpflug Placido corneal topographer. *Journal of Cataract and Refractive Surgery*.

[10] Savini G., Carbonelli M., Barboni P., Hoffer K. J. (2011). Repeatability of automatic measurements performed by a dual Scheimpflug analyzer in unoperated and post-refractive surgery eyes. *Journal of Cataract and Refractive Surgery*.

[11] Guilbert E., Saad A., Grise-Dulac A., Gatinel D. (2012). Corneal thickness, curvature, and elevation readings in normal corneas: combined Placido-Scheimpflug system versus combined Placido-scanning-slit system. *Journal of Cataract and Refractive Surgery*.

[12] Savini G., Carbonelli M., Sbreglia A., Barboni P., Deluigi G., Hoffer K. J. (2011). Comparison of anterior segment measurements by 3 Scheimpflug tomographers and 1 Placido corneal topographer. *Journal of Cataract and Refractive Surgery*.

[13] Lattimore M. R., Kaupp S., Schallhorn S., Lewis R. (1999). Orbscan pachymetry: implications of a repeated measures and diurnal variation analysis. *Ophthalmology*.

[14] Bland J. M., Altman D. G. (1996). Measurement error. *British Medical Journal*.

[15] Bland J. M., Altman D. G. (1986). Statistical methods for assessing agreement between two methods of clinical measurement. *The Lancet*.

[16] Spadea L., Giammaria D., Di Genova L., Fiasca A. (2007). Comparison of optical low coherence reflectometry and ultrasound pachymetry in the measurement of central corneal thickness before and after photorefractive keratectomy. *Journal of Refractive Surgery*.

[17] Su P.-F., Lo A. Y., Hu C.-Y., Chang S.-W. (2008). Anterior chamber depth measurement in phakic and pseudophakic eyes. *Optometry and Vision Science*.

[18] Hernández-Camarena J. C., Chirinos-Saldaña P., Navas A. (2014). Repeatability, reproducibility, and agreement between three different Scheimpflug systems in measuring corneal and anterior segment biometry. *Journal of Refractive Surgery*.

[19] Maresca N., Zeri F., Palumbo P., Calossi A. (2014). Agreement and reliability in measuring central corneal thickness with a rotating Scheimpflug-Placido system and ultrasound pachymetry. *Contact Lens and Anterior Eye*.

[20] Huang J., Ding X., Savini G. (2013). A comparison between scheimpflug imaging and optical coherence tomography in measuring corneal thickness. *Ophthalmology*.

[21] Sedaghat M. R., Daneshvar R., Kargozar A., Derakhshan A., Daraei M. (2010). Comparison of central corneal thickness measurement using ultrasonic pachymetry, rotating scheimpflug camera, and scanning-slit topography. *American Journal of Ophthalmology*.

[22] O'Donnell C., Maldonado-Codina C. (2005). Agreement and repeatability of central thickness measurement in normal corneas using ultrasound pachymetry and the OCULUS Pentacam. *Cornea*.

[23] Al-Mohtaseb Z. N., Wang L., Weikert M. P. (2013). Repeatability and comparability of corneal thickness measurements obtained from Dual Scheimpflug Analyzer and from ultrasonic pachymetry. *Graefe's Archive for Clinical and Experimental Ophthalmology*.

[24] Chen S., Huang J., Wen D., Chen W., Huang D., Wang Q. (2012). Measurement of central corneal thickness by high-resolution Scheimpflug imaging, Fourier-domain optical coherence tomography and ultrasound pachymetry. *Acta Ophthalmologica*.

[25] Al-Mezaine H. S., Al-Amro S. A., Kangave D., Sadaawy A., Wehaib T. A., Al-Obeidan S. (2008). Comparison between central corneal thickness measurements by oculus pentacam and ultrasonic pachymetry. *International Ophthalmology*.

[26] Lackner B., Schmidinger G., Pieh S., Funovics M. A., Skorpik C. (2005). Repeatability and reproducibility of central corneal thickness measurement with pentacam, orbscan, and ultrasound. *Optometry and Vision Science*.

[27] Jahadi Hosseini H. R., Katbab A., Khalili M. R., Abtahi M. B. (2010). Comparison of corneal thickness measurements using Galilei, HR Pentacam, and ultrasound. *Cornea*.

[28] Jorge J., Rosado J. L., Díaz-Rey J. A., González-Méijome J. M. (2013). Central corneal thickness and anterior chamber depth measurement by Sirius Scheimpfug tomography and ultrasound. *Clinical Ophthalmology*.

[29] Mukhopadhyay D. R., North R. V., Hamilton-Maxwell K. E. (2011). Effect of a proparacaine 0.50%-sodium fluorescein 0.25% mix and contact ultrasound pachymetry on central and midperipheral corneal thickness measured by noncontact optical pachymetry. *Journal of Cataract and Refractive Surgery*.

